# Pharmaceuticals: A Headache for Water Treatment

**Published:** 2006-05

**Authors:** Adrian Burton

Acetaminophen is turned into at least two toxic compounds by chlorination treatment, researchers report in the 15 January 2006 issue of *Environmental Science & Technology*, raising concerns about the fate of this and other pharmaceuticals that end up in water supplies. Acetaminophen is one of the most widely used over-the-counter painkillers in the world—in the United States alone, some 37,000 metric tons are produced each year, says coauthor Mary Bedner, a research chemist at the National Institute of Standards and Technology. “Some of this is reaching the environment,” she says, “but no one really knows what happens to it or what effect it might ultimately have on ecosystems or people.”

Reports of acetaminophen in European rivers have appeared since the 1990s, and in the 15 March 2002 issue of *Environmental Science & Technology* a USGS team reported detecting it in nearly a quarter of the water bodies it sampled. “It gets there through wastewater [i.e., via human excretion] and in some cases through poor disposal practices,” says Nick Voulvoulis, a senior lecturer in natural sciences at Imperial College London. Only 22% of Britons and just 1.4% of Americans return unwanted medicines to pharmacies, says Voulvoulis. More than 35% of U.S. nonreturners flush unused drugs down the toilet, while most British drugs end up in landfills, from which they can leach into water bodies.

Concerned that acetaminophen’s structure renders it likely to react with chlorine, Bedner and colleague William MacCrehan used reversed-phase liquid chromatography to follow its interaction with the chlorinating agent hypochlorite. Under simulated treatment conditions in samples of distilled water and wastewater, 11 new compounds were formed from acetaminophen within an hour, the time the reactants would likely be in contact at any plant. Among them were 1,4-benzoquinone (a mutagen) and *N*-acetyl-*p*-benzoquinone imine (a hepatotoxicant also produced during acetaminophen metabolism that is responsible for overdose deaths). Together, these compounds represented the fate of nearly 27% of the original drug concentration.

“Fortunately, these are unstable compounds, especially in the presence of sulfite, which is sometimes used to dechlorinate treated water, so they are unlikely to persist long in the environment,” Bedner says. “However, they could accumulate where treated wastewater is returned to rivers, and the effects of resupply over long periods are unknown.” The results also raise the question of what other drug-derived toxicants are out there, she says.

“This work shows we need to know much more about the fate of the drugs that contaminate our water supplies,” says Damià Barceló, a professor of environmental chemistry at Barcelona’s Centre for Research and Development. “We also have to look for what they turn into. Searching only for the original compounds themselves will not reveal all the dangers these contaminants may pose.”

## Figures and Tables

**Figure:**
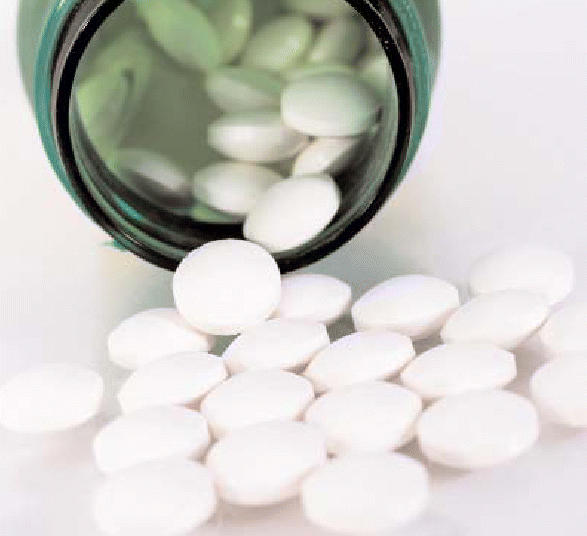
Remedy or pain? The presence of toxic metabolites in water supplies makes you wonder.

